# An assessment of nutritional status in children of rural, northern KwaZulu-Natal province

**DOI:** 10.4102/safp.v62i1.5040

**Published:** 2020-05-21

**Authors:** Kelly R. Gate, Nompumelelo G. Mfeka-Nkabinde, Kantharuben Naidoo

**Affiliations:** 1 Department of Family Medicine, University of KwaZulu-Natal, Durban, South Africa

**Keywords:** malnutrition, overnutrition, undernutrition, severe acute malnutrition, moderate acute malnutrition, stunting, wasting, thinness, overweight

## Abstract

**Background:**

Childhood malnutrition in South Africa is largely perceived as one of undernutrition, with the opposite end of the spectrum (overnutrition) being evidenced in the increasing prevalence of childhood obesity, demonstrated to be associated with chronic metabolic diseases in adulthood. Targeting childhood malnutrition is a potential interventional strategy to prevent non-communicable diseases amongst adults. As the prevalence of malnutrition (undernutrition and overnutrition) in rural, northern KwaZulu-Natal province, South Africa, is largely unknown, this study aimed to determine the baseline nutritional status of children attending primary healthcare facilities within the Bethesda Hospital catchment area.

**Methods:**

This quantitative, cross-sectional study included children aged 6 weeks to 19 years, attending any primary healthcare clinics for over a 3 months period. Anthropometric measurements were obtained to categorise the children according to the World Health Organisation’s (WHO) nutritional classifications.

**Results:**

Stunting in children aged less than 5 years was found to be lower (14%) than nationally representative studies (27%); however, 14.4% of the infants aged 6 weeks to 5 months were overweight, increasing to 32.3% in those aged 14–19 years. Males in the 6-weeks to 5-month age group were more likely to be overweight/obese and stunted than females in the same age group.

**Conclusion:**

Undernutrition is showing a downward trend, which is a testament to initiatives to reduce food insecurity amongst the poor. However, the emerging upward trend of overweight/obesity in children of all ages, indicates the need to have a national discussion on over- and undernutrition, its causes and implications.

## Introduction

South Africa is currently experiencing a complex health transition and has been described as a country with both undernutrition and overnutrition.^1^ The dynamics of these seemingly contradictory conditions are complex and diverse, both being dependent on various social, economic and geographical factors, in addition to society’s changing culture and norms.^2^

Malnutrition is defined by the World Health Organisation (WHO) as ‘the cellular imbalance between the supply of nutrients and energy and the body’s demand for them to ensure growth, maintenance and specific functions’.^3^ This definition not only refers to undernutrition (wasting and stunting) but also encompasses both ends of the spectrum, that is, undernutrition and overnutrition.^4^ Refer to Appendix 1 for more definitions of terminologies used within this article.

According to the 2013 South African National Health and Nutrition Survey 1 (SANHANES-1) report, undernutrition, although improving, is still a problem in South Africa with 26.5% of children aged 1–3 years stunted, 2.2% being wasted and 6.1% of them being underweight.^5^ The effects of malnutrition not only increase the risk of morbidity and mortality during childhood and adulthood but also increase the incidence of chronic diseases such as abnormal lipid profile, diabetes, hypertension and cardiovascular disease later in life.^6^ This association was found to be more pronounced in low- and middle-income countries, which account for half of global population.^6^

Malnutrition also affects the cognitive ability of child and is also negatively associated with potential earning capacity in later life.^6^ Rapid weight gain in late childhood and adolescence following a period of undernutrition during the formative years may represent a worst-case scenario for cardiovascular and metabolic diseases.^6^ This association between childhood malnutrition and negative effects on adult health outcomes is known as the ‘thrifty gene’ or Barker hypothesis.^7^ The largest proportion of stunted children are found in African countries, affecting 35.6% of those aged less than 5 years,^7^ with 27% of those under the age of 5 years being affected in South Africa.^8^

However, sub-Saharan Africa (SSA) is one of the few places where the prevalence of stunting in children, despite interventions, remains stagnant, whilst obesity in this population continues to increase.^9^

In the South African 2005 National Food Consumption Survey Fortification Baseline (NFCS: FB-I), 10% of children aged 1–9 years were found to be overweight, whilst 4% were classified as obese.^10^ This placed obesity as the second most common adverse anthropometric outcome in children.^11^ The short-term consequences of obesity on a child’s psychological and cardiovascular well-being, as well as other adverse health effects, are well recognised.^12^ There are also the adverse effects on health in adulthood, in particular, metabolic syndrome and premature mortality.^12^ Studies confirm that children in some rural areas of South Africa are following global trends of childhood obesity as demonstrated in the Northwest province.^13^ Targeting childhood malnutrition is therefore a potential interventional strategy to prevent health complications in children and non-communicable diseases (NCDs) amongst adults.

This study therefore aimed to determine the baseline nutritional status of children aged 6 weeks–19 years attending primary healthcare (PHC) facilities within the Bethesda Hospital catchment area of rural, northern KwaZulu-Natal (KZN) province, South Africa. The study had the following two objectives:

- To obtain anthropometric measurements of children aged 6 weeks–19 years attending PHC facilities.
- To determine the rates of malnutrition and compare these with national malnutrition trends.

## Method

The study was conducted within the municipal boundary of Jozini Municipality, uMkhanyakude district, KZN, South Africa. The district is located in the far north region of KZN, has a population of 638 011 with a population density of 46.0 persons per km^2^, making it the most sparsely populated district in the province.^14^ Only 13.4% of households have pipe-borne water inside their dwellings, 38.4% use electricity for lighting and only 9.0% have weekly refuse removal services.^14^ Of the population’s citizens who are aged 20 years and older, 25.4% have obtained a matric certificate and 4.9% have a higher education.^14^ The unemployment rate is high at 42.8% and the district falls within socio-economic quintile 1, amongst the poorest districts in South Africa.^14^ In spite of the deprived status of the district, it performed very well with respect to certain health indicators, being amongst the 10 best performing districts in the country.^14^ Bethesda Hospital is one of the five public sector rural, district hospitals and services a network of eight PHC clinics.

The study population comprised children aged 6 weeks–19 years attending PHC facilities within the Bethesda Hospital catchment area, with those presenting during a defined period (11 April 2018 – 27 July 2018) being eligible to participate. All non-pregnant children who were willing to consent, and whose adult caregiver signed consent/assent, were included. Children were categorised based on mid-upper arm circumference (MUAC) appropriateness and/or WHO age classifications into the following five groups: 6 weeks to 5 months, 6 months to 4 years, 5–9 years, 10–13 years and 14–19 years. The WHO classifies children’s nutritional status under five different categories, from children aged 5 years and older, and therefore the data for this study were collected accordingly to remain in line with the WHO definitions and nutritional classifications. Children younger than 6 weeks of age were also excluded, as routine growth monitoring at PHC level only commences at the scheduled 6-week Extended Programme on Immunization (EPI) visit. Critically ill children requiring emergency care were not included in this study.

Anthropometric measurements were taken from all children, fulfilling the inclusion criteria, by trained research assistants and nutritional advisors in accordance with the WHO guidelines.^15^

The classification tables of weight for length for participants less than 5 years old or less than 100 cm in length/height were obtained from the Community Management of Acute Malnutrition (CMAM) guidelines, and the body mass index (BMI) adjusted for age for participants aged 5–19 years were obtained from relevant WHO guidelines.^16,17^ Any child found to be having severe acute malnutrition (SAM), moderate acute malnutrition (MAM), stunted, thin, very thin, overweight or obese was appropriately referred to a PHC nurse or doctor for further management.

The following variables were recorded according to the WHO or other standards guidelines:

- Age: Dates of birth and assessment were entered into an Excel spread sheet and the age calculated in years, months, days using the ‘datediff’ function. If the child was born prematurely, then the corrected/adjusted age was utilised.
- Gender: Self-reported by child or caregiver.
- Ethnic group: Self-reported by child or caregiver.
- Weight: Measured with an appropriate calibrated scale. Children unable to stand and under 20 kg were measured with a calibrated electronic baby scale. Those weighing more than 20 kg and were unable to stand were measured whilst their caretaker held them, from which the caretaker’s weight was subtracted, that is, tared weight.
- Length/height: Measured with a stadiometer, calibrated in centimetres, for children aged more than 2 years or if they were taller than 87 cm. Children aged less than 2 years, had a length less than 87 cm or were unable to stand, had their recumbent length measured using a measuring board or infantometer.^15^
- Mid-upper arm circumference: Measured with a tape measure, with the midpoint between the acromion process and olecranon of the left arm being identified around which the tape measure was placed circumferentially.^18^ The MUAC measurement was then read through the ‘window’ on the MUAC tape to the nearest 0.1 cm.
- Body mass index: Calculated for all those aged 5–19 years based on weight and height for age and gender.

## Statistical analysis

The data were entered in Excel, with IBM SPSS version 25 being used for analysis. For objective 1, the data were analysed using the classification of weight and height according to the 2007 WHO growth standards with the WHO Anthro 2011 program (version 3.2.2).^19^ To determine the rate of malnutrition (objective 2), the *Z*-scores were determined using WHO’s 2007 software, Anthro for those aged 6 weeks to 4 years and AnthroPlus for those aged 5–19 years.^20^

Participants with *Z*-scores above +5 standard deviation (SD) or below −5 SD were considered as outliers and excluded from the analysis. Pearson’s chi-square tests were used to compare age and sex groups regarding body image classifications, with a *p*-value of < 0.05 being regarded as statistically significant.

### Ethical considerations

Approval to conduct the study was obtained from the Biomedical Research Ethics Committee (BREC), University of Kwazulu-Natal (reference number: BE24/17).

## Results

During the study period, the anthropometry of 700 children aged 6 weeks to 19 years was measured (objective 1), with the 6-month to 4-year cluster representing the largest proportion (Table 1). This can be explained by the many children in this age group attending clinics for the ‘Well Baby’/EPI. All children were of African ethnicity, and more were females (*n* = 370) than males (*n* = 330).

**TABLE 1:** Frequencies within each age cluster.

Cluster	Frequency	Percentage
6 weeks–5 months	188	26.9
6 months–4 years	379	54.1
5–9 years	67	9.6
10–13 years	35	5.0
14–19 years	31	4.4
**Total**	**700**	**100.0**

In the under 5-year olds, the majority (82.4%) of children were in the acceptable weight category, whilst 11.8% were overweight, 3.7% were obese and only 0.02% were undernourished (SAM or MAM) as further detailed in Table 2.

**TABLE 2:** Nutritional and height status of children aged less than 5 years.

Variable	6 weeks–5 months		6 months–4 years		Total	
	%	*n*	%	*n*	%	*n*
**Nutritional classification**						
SAM	0.5	1	0.0	0	0.2	1
MAM	0.0	0	2.9	11	1.9	11
Acceptable	79.3	149	83.9	318	82.4	467
Overweight	14.4	27	10.6	40	11.8	67
Obese	5.9	11	2.6	10	3.7	21
Total	100.0	188	100.0	379	100.0	567
**Height**						
Acceptable	-	-	86.0	326	86.0	326
Stunted	-	-	14.0	53	14.0	53
Total	-	-	100.0	379	100.0	379

SAM, severe acute malnutrition; MAM, moderate acute malnutrition.

In the height classification (Table 2), 14% of children aged under 5 years in the study cohort were stunted.

In the category of children aged 5 years and above (Table 3), it was found that although most children were within the acceptable nutritional category, 6.0% of those aged 5–9 years were severely thin, whilst 32.3% of children aged 14–19 years were overweight with 3.2% in this age group being classified as obese.

**TABLE 3:** Nutritional classification in children aged 5 years and above.

Cluster (age)	Severely thin		Thin		Acceptable		Overweight		Obese		Total	
	%	*n*	%	*n*	%	*n*	%	*n*	%	*n*	%	*n*
5–9 years	6.0	4	4.5	3	82.1	55	6.0	4	1.5	1	100.0	67
10–13 years	0.0	0	11.4	4	80.0	28	8.6	3	0.0	0	100.0	35
14–19 years	3.2	1	6.5	5	54.8	17	32.3	10	3.2	1	100.0	31
**Total**	**3.8**	**5**	**6.8**	**9**	**75.2**	**100**	**12.8**	**17**	**1.5**	**2**	**100.0**	**133**

In children aged 5 years and above (Table 4), the prevalence of malnutrition shows the majority, with all age bands being classified as acceptable; however, 5.4% of females and 6.7% of males aged 5–9 years were classified as severely thin, and females aged 14–19 years were overweight (32.3%) or obese (3.2%). Boys aged 5–13 years were more likely to be classified as severely thin/thin than females of the same age.

**TABLE 4:** Nutritional classification according to gender in children aged 5 years and older.

Cluster (age)	Severely thin		Thin		Acceptable		Overweight		Obese		Total	
	%	*n*	%	*n*	%	*n*	%	*n*	%	*n*	%	*n*
**Females**												
5–9 years	5.4	2	0.0	0	89.2	33	5.4	2	0.0	0	100.0	37
10–13 years	0.0	0	9.5	2	76.2	16	14.3	3	0.0	0	100.0	21
14–19 years	0.0	0	7.7	2	53.8	14	34.6	9	3.8	1	100.0	26
Total	2.4	2	4.8	4	75.0	63	16.7	14	1.2	1	100.0	84
**Males**												
5–9 years	6.7	2	10.0	3	73.3	22	6.7	2	3.3	1	100.0	30
10–13 years	0.0	0	14.3	2	85.7	12	0.0	0	0.0	0	100.0	14
14–19 years	20.0	1	0.0	0	60.0	3	20.0	1	0.0	0	100.0	5
Total	6.1	3	10.2	5	75.5	37	6.1	3	2.0	1	100.0	49

Regarding the nutritional classifications of children aged 5 years and older (Table 4), there was prevalence of overweight amongst adolescents aged 14–19 years (females 34.6%, males 20%) with an emerging 3.2% of obesity amongst females in the same age group.

## Discussion

Whilst a number of surveys across South Africa have highlighted the rate of severe malnutrition in children aged under 5 years, there is little current data for those under the age of 19 years living in the rural settings of South Africa.

In this study, male children aged 6 weeks to 5 months were more likely to be overweight or obese than female children, and were classified as stunted. Male children have been noted to be more commonly classified as malnourished in comparison to females in multiple SSA studies.^10,11^ The mechanism of this occurrence is largely unknown, but it is thought to be related to boys suffering more from environmental stress than girls.^1,10,21^

The South African Demographic and Health Survey Report of 2016 estimated that in children under the age of 5 years, 3% were wasted, 6% were underweight and 27% were stunted.^8^ Lower rates of stunting (14% vs. 26.5%), severe wasting (0.002% vs. 2.2%) and underweight (0.2% vs. 6.1%) in those aged 6 weeks–4 years were found in our study. However, both studies found similar rates of overweight children in the same age group (11.8% vs. 13%).^8^

In this study, 14.4% prevalence of overweight in children aged 6 weeks to 5 months is a cause for concern. According to the District Health Barometer 2016/2017, the national average breastfeeding rate (at the 3rd Hepatitis B dose) was 41.6%, this being below the national target of 55%.^22^ The early introduction of complementary feeding with low nutritional value is common in South Africa^10^ and combined with prevailing low national breastfeeding rates may contribute to the increased rates of overweight and obesity in children in this age group.^22^

According to the District Health Barometer of 2013/2014, uMkhanyakude district’s acute malnutrition rate in children aged under 5 years was 6.4 per 1000.^14^ The Annual Department of Health report indicates that the prevalence of SAM, the most severe form of malnutrition, has shown a downward trend within uMkhanyakude, with 232 related admissions for the period 2016/2017 versus 159 for 2017/2018.^23^ This could be because of many intervention programmes of National Department of Health, one being the Integrated Nutrition Programme (INP), which was initiated with the aim of targeting vulnerable groups and communities, specifically children under the age of 5 years, to improve early childhood nutrition.^24^

Both ends of the malnutrition spectrum have potential adverse consequences for a child in later life.^6^ These include increased risk of both infection in underweight children and chronic diseases of lifestyles, such as diabetes, in overweight/obese children.^25^ Although the majority of children in this study older than 5 years were found to be in an acceptable weight range, the emerging rate (32.3%) of overweight/obesity within the age group of 14–19 years is of concern. This high rate of childhood obesity is consistent with findings in the other rural areas of South Africa.^10,13^ As children become older, the trend is for female children to more likely be overweight or obese as they progress through the late adolescent phase.^1,26^ This finding is corroborated by Shisana et al. in the SANHANES-1 report, which showed that girls, after the age of 5 years, were more likely to be ‘heavier’ than boys.^5^ According to Dietz, the prevalence of obesity in children and adolescents would exceed the prevalence of undernutrition by 2022.^27^ An increase in gross domestic product (GDP), changes in food consumption and decreased physical activity have been listed as causes of the increase observed in childhood obesity rates.^27^ A commonly seen characteristic of nutrition transition, which South Africa is currently experiencing, is the increase in intake of energy-dense foods that have higher sugar, fat, oil and animal-derived products.^26^ The impact of this could be responsible for the 32% overweight prevalence in this study.

Undernutrition remains a concern within the uMkhanyakude district, but its significant decreased prevalence amongst those attending clinic in this study suggests that the multiple initiatives that are currently in place are addressing this end of the malnutrition spectrum. Similar to many SSA countries, the emerging upward trend of overweight and obesity amongst children and adolescents is a concern, which needs focused and target-specific initiatives to prevent it detrimentally affecting their health and already increasing the existing large adult population with NCDs. The treatment and prevention of obesity in children within the South African context requires a multi-sectoral approach.^28^ Stakeholders need to fully understand the reasons for obesity within rural and urban communities and to implement community-specific interventions to address it.^28^ More research is needed into the factors that result in children being exposed to lifestyles that result in their being obese, and what needs to be carried out to reduce the prevalence. These initiatives should be implemented as early as possible in a child’s life to prevent malnutrition and the onset of obesity.^28^

### Limitations of the study

A number of limitation are acknowledged, with the data showing that MUAC is age-dependent, which could result in the over-diagnosis of wasting amongst younger children and its under-diagnosis amongst older children when a fixed cut-off value is used.^29^

Body mass index is also not without its limitations in children and should be interpreted with caution. Although it is widely used as a surrogate measure of adiposity, it is a measure of increased weight relative to height, rather than excess body fat.^30^ The interpretation of BMI amongst children and adolescents is further complicated by the changes that occur in weight, height and body composition during the typical phases of childhood growth.^30^ Whilst a high BMI-for-age is a good indicator of excess fat mass, amongst thinner children it can be largely because of fat-free mass, which is typically more common in boys, especially during later adolescence.^30^

It could be argued that children attending PHC facilities do so because they are ill, which could adversely affect their nutritional status and increase the apparent incidence of malnutrition. This could be particularly true for children aged over 5 years, who no longer are part of EPI. Conversely, it could be argued that children who are not attending clinic, because of various social and economic factors, are more at risk for malnutrition and may have been missed in this study. Children are also less likely to attend clinics for routine medical care during their adolescent years, which resulted in the low numbers of these age groups enrolling in this study. The results of this study would need to be generalised to the broader population of the area with extreme caution, with a much larger epidemiological study required to assess the nutritional status of children within the study area.

Furthermore, the cause or associations of malnutrition were not determined, requiring further research for effective strategies to be developed and implemented.

## Conclusion

It is evident from the results of this study that efforts to curb childhood undernutrition appear to be bearing fruit. However, even in rural areas of South Africa, residents are following the global trend of increasing childhood obesity. Both undernutrition and overnutrition could have serious short-term and long-term consequences for children in terms of cognitive and health effects. Understanding why children do not receive the right nutrition is essential if they are to enjoy a good quality of life, develop their full potential, and be constructive and contributing citizens.

## Appendix 1: Definitions

Adult: Any person more than 19 years of age as defined by the World Health Organisation (WHO). Adolescent: Persons aged 10–19 years (inclusive), being the period of human growth and development that occurs after childhood and before adulthood, as defined by the WHO. Body mass index (BMI): A measure of weight adjusted for height being calculated as weight in kilograms divided by the square of height in metres. Child: Any person 19 years of age and below (less than 20 years of age) as defined by the WHO. Infant: Any child who has not yet had his/her first birthday, as defined by the WHO. Mid-upper arm circumference (MUAC): An anthropometric measurement used in the nutritional assessment of children aged 6 months or older. It is the circumference of the upper arm at the midpoint between an imaginary line drawn from the acromion to the olecranon. Moderate acute malnutrition (MAM): Defined by moderate wasting (low weight-for-height/ length between -2 and -3 standard deviation [SD]) or MUAC between 11.5 cm and 12.4 cm.^4^ Page 2 of 7 Original Research http://www.safpj.co.za Open Access Malnutrition: The cellular imbalance between the nutrients and energy supply and the body’s demands to ensure growth, maintenance and specific functions. Neonate: First 28-day period of life as defined by the WHO. Obesity: More than 3 SD weight-for-height median of the National Center for Health Statistics (NCHS)/WHO international reference for children aged under 5 years and more than 2 SD BMI-for-age and sex in children aged over 5 years (equivalent to BMI 30 kg/m^2^ at 19 years). Overnutrition: The excess intake of nutrients and energy that cannot be expended results in overweight and obesity. Overweight: More than 2 SD weight-for-height median of the NCHS/WHO international reference for children aged under 5 years and more than 1 SD BMI-for-age and sex in children aged over 5 years (equivalent to BMI 25 kg/m2 at 19 years). Severe acute malnutrition (SAM): The severest form of undernutrition, defined by the presence of bilateral pitting pedal oedema or severe wasting (very low weight for length/ height less than −3 SD) or MUAC <11.5 cm (in children aged 6–59 months), being associated with other clinical signs such as poor appetite. Severe Thinness: More than 3 SD below BMI-for-age and sex in children aged over 5 years.^6^ Stunting: More than 2 SD below the international reference median value of height/length-for-age.^6^ Thinness: More than 2 SD BMI-for-age and sex in children aged over 5 years. Undernutrition: The outcome of insufficient food intake and repeated infectious diseases, and includes being underweight for age, stunted, wasted and micronutrient malnutrition. Underweight: More than 2 SD below the international reference median value of weight-for-age. Wasting: Loss of weight because of inadequate nutrient intake with more than 2 SD below the international reference median value of weight-for-height in children aged under 5 years. Z-score: The deviation of an individual’s value from the median value of a reference population divided by the SD of the reference population.

## References

[1] Reilly JJ, Methven E, McDowell ZC, et al. Health consequences of obesity. Arch Dis Child. 2003;88(9):748–752. 10.1136/adc.88.9.748 12937090 PMC1719633

[2] Mehta NM, Corkins MR, Lyman B, et al. Defining pediatric malnutrition: A paradigm shift toward etiology-related definitions. JPEN J Parenter Enteral Nutr. 2013;37(4):460–481. 10.1177/0148607113479972 23528324

[3] Kimani-Murage EW. Exploring the paradox: Double burden of malnutrition in rural South Africa. Glob Health Action. 2013;6:19249. 10.3402/gha.v6i0.19249 23364082 PMC3556706

[4] De Onis M, Branca F. Childhood stunting: A global perspective. Matern Child Nutr. 2016;12(Suppl 1):12–26. 10.1111/mcn.12231 27187907 PMC5084763

[5] Eckholm E, Record F. The two faces of malnutrition. Worldwatch paper (USA). Washington, DC: International Information System for the Agricultural Science and Technology (AGRIS). Washington DC; 1976.

[6] Shisana O, Labadarios R, Rehle T, et al. South African national health and nutrition examination survey (SANHANES-1). Cape Town: HSRC Press; 2013.

[7] Victora CG, Adair L, Fall C, et al. Maternal and child undernutrition: Consequences for adult health and human capital. Lancet. 2008;371(9609):340–357. 10.1016/S0140-6736(07)61692-4 18206223 PMC2258311

[8] Reinhardt K, Fanzo J. Addressing chronic malnutrition through multi-sectoral, sustainable approaches: A review of the causes and consequences. Front Nutr. 2014;1:13. 10.3389/fnut.2014.00013 25988116 PMC4428483

[9] National Department of Health (NDoH), STATS SA (SSASS), South African Medical Research, Council (SAMRC), ICF. South Africa demographic and health survey 2016. Pretoria: NDoH, Stats SA, SAMRC and ICF; 2016.

[10] Keino S, Plasqui G, Ettyang G, Van den Borne B. Determinants of stunting and overweight among young children and adolescents in sub-Saharan Africa. Food Nutr Bull. 2014;35(2):167–178. 10.1177/156482651403500203 25076764

[11] Lesiapeto MS, Smuts CM, Hanekom SM, Du Plessis J, Faber M. Risk factors of poor anthropometric status in children under five years of age living in rural districts of the Eastern Cape and KwaZulu-Natal provinces, South Africa. S Afr J Clin Nutr. 2010;23(4):202–207. 10.1080/16070658.2010.11734339

[12] Steyn N, Labadarios D, Maunder E, Nel J, Lombard C, Survey Dot NFC. Secondary anthropometric data analysis of the National Food Consumption Survey in South Africa: The double burden. Nutrition. 2005;21(1):4–13. 10.1016/j.nut.2004.09.003 15661473

[13] Pienaar AE. Prevalence of overweight and obesity among primary school children in a developing country: NW-CHILD longitudinal data of 6–9-yr-old children in South Africa. BMC Obes. 2015;2(1):2. 10.1186/s40608-014-0030-4 26217517 PMC4511440

[14] Massyn N, Day C, Peer N, Padarath A, Barron P, English R. District health barometer 2013/2014. Durban: Health Systems Trust; 2014.

[15] World Health Organisation (WHO). Training course on child growth assessment. Geneva: WHO; 2008.

[16] World Health Organisation (WHO). Growth reference data for 5–19 years. Geneva: WHO; 2007.

[17] World Health Organisation (WHO). Guideline: Updates on the management of severe acute malnutrition in infants and children. Geneva: WHO; 2013. 24649519

[18] United Nations International Children’s Emergency Fund (UNICEF). Measuring MUAC [homepage on the Internet]. [cited 2017 Feb 1]. Available from: http://www.unicef.org/nutrition/training/3.1.3/1.html

[19] World Health Organisation (WHO). WHO anthro survey analyser 2005, Beta version, 17 February 2006: Software for assessing growth and development of world’s children. Geneva: WHO; 2006.

[20] World Health Organisation (WHO). WHO anthroPlus for personal computers manual: Software for assessing growth of world’s children and adolescents [homepage on the Internet]. Geneva: World Health Organisation; 2009. [cited 2017 Feb 1]. Available from: http://www.who.int/growthref/tools/en/

[21] Hien N, Kam S. Nutritional status and the characteristics related to malnutrition inchildren under five years of age in Nghean, Vietnam. J Prev Med Public Health. 2008;41(4):232–240. 10.3961/jpmph.2008.41.4.232 18664729

[22] Massyn N, Padarath A, Peer N, Day C. District health barometer 2016/17. Durban: Health Systems Trust; 2017.

[23] Health K-NDo. Annual report 2017/18. 2018; Pietermaritzburg: Department of Health, Kwazulu-Natal.

[24] Iversen PO, Marais D, Du Plessis L, Herselman M. Assessing nutrition intervention programmes that addressed malnutrition among young children in South Africa between 1994–2010. Afr J Food Agric Nutr Dev. 2012;12(2):4. 10.1016/S0140-6736(17)32129-3

[25] NCD Risk Factor Collaboration (NCD-RisC) (1040 collaborators). Worldwide trends in body-mass index, underweight, overweight, and obesity from 1975 to 2016: A pooled analysis of 2416 population-based measurement studies in 128·9 million children, adolescents, and adults. Lancet. 2017;390(10113):2627–2642. 29029897 10.1016/S0140-6736(17)32129-3 PMC5735219

[26] Kimani-Murage EW, Kahn K, Pettifor JM, Tollman SM, Dunger DB, Gómez-Olivé XF. The prevalence of stunting, overweight and obesity, and metabolic disease risk in rural South African children. BMC Public Health. 2010;10:158. 10.1186/1471-2458-10-158 20338024 PMC2853509

[27] Dietz WH. Double-duty solutions for the double burden of malnutrition. Lancet. 2017;390(10113):2607–2608. 10.1016/S0140-6736(17)32479-0 29029895

[28] Kruger H, Puoane T, Senekal M, Van der Merwe M. Obesity in South Africa: Challenges for government and health professionals. Public Health Nutr. 2005;8(5):491–500. 10.1079/PHN2005785 16153330

[29] De Onis M, Yip R, Mei Z. The development of MUAC-for-age reference data recommended by a WHO expert committee. Bull World Health Org. 1997;75(1):11. 10.1177/156482659701800204 9141745 PMC2486977

[30] Freedman DS, Wang J, Maynard LM, et al. Relation of BMI to fat and fat-free mass among children and adolescents. Int J Obes. 2005;29(1):1–8. 10.1038/sj.ijo.0802735 15278104

